# Preferential and selective degradation and removal of amelogenin adsorbed on hydroxyapatites by MMP20 and KLK4 *in vitro*

**DOI:** 10.3389/fphys.2014.00268

**Published:** 2014-07-24

**Authors:** Li Zhu, Haichuan Liu, H. Ewa Witkowska, Yulei Huang, Kataro Tanimoto, Wu Li

**Affiliations:** ^1^Department of Orofacial Sciences, School of Dentistry, University of California, San FranciscoSan Francisco, CA, USA; ^2^Department of Obstetrics, Gynecology and Reproductive Sciences, University of California, San FranciscoSan Francisco, CA, USA; ^3^Department of Oral Medicine, Guanghua School of Stomatology, Sun Yat-sen UniversityGuangdong, China; ^4^Departments of Orthodontics and Craniofacial Developmental Biology, Hiroshima University Graduate School of Biomedical SciencesHiroshima, Japan

**Keywords:** amelogenin, tooth enamel, MMP20, KLK4, protein interactions, hydroxyapatites, biomineralization, proteolysis

## Abstract

The hardest tooth enamel tissue develops from a soft layer of protein-rich matrix, predominated by amelogenin that is secreted by epithelial ameloblasts in the secretory stage of tooth enamel development. During enamel formation, a well-controlled progressive removal of matrix proteins by resident proteases, Matrix metalloproteinase 20 (MMP20), and kallikrein 4 (KLK4), will provide space for the apatite crystals to grow. To better understand the role of amelogenin degradation in enamel biomineralization, the present study was conducted to investigate how the adsorption of amelogenin to hydroxyapatite (HAP) crystals affects its degradation by enamel proteinases, MMP20 and KLK4. Equal quantities of amelogenins confirmed by protein assays before digestions, either adsorbed to HAP or in solution, were incubated with MMP20 or KLK4. The digested samples collected at different time points were analyzed by spectrophotometry, SDS-PAGE, high performance liquid chromatography (HPLC), and LC-MALDI MS/MS. We found that majority of amelogenin adsorbed on HAP was released into the surrounding solution by enzymatic processing (88% for MMP20 and 98% for KLK4). The results show that as compared with amelogenin in solution, the HAP-bound amelogenin was hydrolyzed by both MMP20 and KLK4 at significantly higher rates. Using LC-MALDI MS/MS, more accessible cleavage sites and hydrolytic fragments from MMP20/KLK4 digestion were identified for the amelogenin adsorbed on HAP crystals as compared to the amelogenin in solution. These results suggest that the adsorption of amelogenin to HAP results in their preferential and selective degradation and removal from HAP by MMP20 and KLK4 *in vitro*. Based on these findings, a new degradation model related to enamel crystal growth is proposed.

## Introduction

The superficial layer of a tooth is enamel, the hardest tissue known in vertebrates. The unique morphological structure and outstanding mechanical properties of the enamel make it different from other mineralized tissues in the body, such as bone, dentin, and cementum. Tooth enamel is comprised mainly of numerous hexagonal carbonated hydroxyapatite (HAP) crystals, organized into rod, and interrod structures with distinct mechanical properties (Ichijo et al., 1992; Plate and Hohling, 1994; Pergolizzi et al., 1995).

The enamel crystals are very thin (20–30 nm in diameter) but extremely long. Many investigators believe that these crystals span the entire thickness of tooth enamel, a distance up to 2.5 mm (Leblond and Warshawsky, 1979; Daculsi et al., 1984; Nanci et al., 1998). The unique morphology and organization of enamel crystals determine its excellent mechanical characteristics while also raise a persistent question of how these enamel crystals form in such a special shape. Solving this puzzle will advance our knowledge of the basic principle of amelogenin-mediated mineralization during enamel development, help us to better understand the fundamental mechanism of an enamel disease, amelogenesis imperfecta, and provide useful information for future acellular tooth enamel regeneration.

The hard enamel tissue develops from a soft protein-rich extracellular matrix secreted by epithelial ameloblasts during the secretory stage of enamel development. At later stages of enamel formation, a well-controlled progressive removal of matrix proteins by resident proteases will provide space for the apatite crystals to grow (Bartlett and Simmer, 1999).

The protein matrix of forming enamel is composed predominantly of amelogenins and its cleavage products (Termine et al., 1980; Fincham et al., 1999). Amelogenin protein is hydrophobic in nature except for its highly charged C-terminus. Amelogenins self-assemble into highly packed and tightly associated nanospheres that interact with apatite crystals to provide the supportive framework for the growth of newly formed crystals with extremely thin and long structure (Robinson et al., 1998; Wen et al., 1999). This intercrystalline protein matrix also serves to prevent premature crystal–crystal fusion during the early stages of enamel formation (Moradian-Oldak et al., 2001).

It has been suggested that amelogenins are critical for the organization of the crystal pattern and regulation of enamel thickness. *In vitro* mineralization studies show that amelogenin can control crystal orientation and growth habit as well as facilitate aggregation of pre-formed hydroxyapatite crystals (Iijima et al., 2002). Mutation of amelogenin can cause amelogenesis imperfecta, a disorder of tooth enamel (Hart et al., 2002; Wright et al., 2003). The essential role of amelogenin in tooth enamel development is also confirmed by the amelogenin-null mice that exhibit defected amelogenesis phenotype with much thinner enamel layers (Gibson et al., 2001).

During enamel mineralization, the step-wise increase in mineral content is concomitant with progressive degradation of amelogenin in the enamel matrix. In particular, during the later stages, amelogenin proteins disappear rapidly and completely to facilitate crystal growth (Fukae and Shimizu, 1974). Matrix metalloproteinase 20 (MMP20) and kallikrein 4 (KLK4) are considered to be the two major amelogenin-processing enzymes during the early and late stages of enamel development, respectively (Bartlett et al., 1996; Bartlett and Simmer, 1999; Simmer and Hu, 2002). However, the relationship between apatite surface, enamel proteases, and enamel crystal growth remains yet to be explored. In this study, we focus on how the adsorption of amelogenin to HAP crystals affects its degradation by enamel proteinases, MMP20 and KLK4. We hypothesize that the binding of amelogenin to apatite may induce protein conformational change and subsequently affect the susceptibility of the adsorbed amelogenin to proteinases. The crystal-protein and protein-proteinase interactions are dynamically coordinated to regulate the mineral accretion. In the present study we have determined the effect of the apatite surface on enamel protease activity and amelogenin cleavage as a means to explain enamel crystal growth through proteolytic amelogenin degradation.

## Materials and methods

### Sample preparation of proteins bound to HAP

Recombinant human amelogenin (rh174) and MMP20 were produced and purified as previously described (Li et al., 1999; Wang et al., 2006). Commercial recombinant human KLK4 was purchased from R&D Systems (Minneapolis, MN, USA). Hydroxyapatite (HAP) was synthesized according to the method reported by Nelson et al. and identified by X-ray diffraction as described in our previous publication (Zhu et al., 2009).

Amelogenin was incubated with HAP crystals at amount over the saturation binding (875 ± 37 μg/m^2^) (Tanimoto et al., 2008a,b; Zhu et al., 2011). After 2 h incubation at room temperature with constant shaking, the protein-HAP complex was centrifuged at 5000 rpm for 5 min. The amount of protein bound was determined by measuring the change in concentration of protein solution before and after binding using Braford assay (BioRad, Hercules, CA).

### Removal of amelogenin from HAP by MMP20 and KLK4 digestion

The digestion of HAP-bound amelogenin (0.2 mg) with MMP20 was performed with enzyme/substrate ratio of 1: 50 (W:W) in 100 μl of reaction buffer (50 mM Tris-HCl, 150 mM NaCl, 10 mM CaCl_2_, 10 μM ZnCl_2_, pH 7.5). At different time points after incubation on an orbital shaker at 37°C, the samples were boiled for 10 min to inactivate MMP20. After centrifugation at 5000 × rpm for 5 min, the supernatant was carefully and completely removed from the HAP sediment. The HAP was dissolved in 10 μl of 0.1% TFA and then reaction buffer was added to bring the volume up to final 100 μl and the pH to neutral. The amount of protein released from HAP during incubation and the amount retained on the mineral were quantitatively determined by UV detection at wavelength 220 nm. Control samples, including HAP-bound amelogenin only and HAP with addition of MMP20 only, were treated in the same manner. MMP20 was replenished at same amounts after 24 h of incubation. The experiments were conducted in triplicate, and the average OD values and standard deviations were calculated. The removal of adsorbed amelogenin by KLK4 was carried out in a buffer with 50 mM Tris-HCl at pH 7.5, 250 mM NaCl and 50 mM CaCl_2_ under the similar conditions and analyzed by an identical methodology as described above.

### Comparative digestion of HAP-bound and HAP-free amelogenin in solution

Same amounts of HAP-bound and unbound amelogenin were digested with MMP20 and KLK4 at enzyme/substrate ratio of 1:50 in equal volumes of their corresponding reaction buffers in a time-course manner. The digestions were carried out at 37°C under continuous shaking. At each time point, 50 μl sample was withdrawn and the hydrolysis was stopped by boiling in a water bath for 10 min. The digested samples were then subjected to SDS-PAGE, reversed-phase high performance liquid chromatography (HPLC), and liquid chromatography matrix-assisted laser desorption ionization tandem mass spectrometry (LC-MALDI MS-MS).

In SDS-PAGE experiments, the digested samples (15 ul for MMP20 or 25 ul for KLK4) were mixed with SDS sample buffer, and resolved by using a 15% SDS-PAGE gel. Prior to HPLC separation, equal volume of 0.1% TFA was added to all the samples to dissolve HAP and release the retained proteins. The HPLC analyses were performed using a C4 column (Varian, Walnut Creek, CA, USA). A linear gradient from 0 to 95% acetonitrile (ACN) in 0.1% TFA was run over a period of 45 min at a flow-rate of 1 ml/min. Elution was monitored via absorbance at 214 nm. The peak areas (mAU/ml) of remaining substrate after hydrolysis were measured and compared.

After 36 h digestion, the samples were centrifuged at 5000 rpm for 5 min. The HAP pellets were dissolved in 50 μl of 0.1% TFA. Both proteolytic fragments still bound to the HAP and those released into the solution were identified by LC-MALDI MS/MS.

### Effect of bare HAP on MMP20 activity

To determine whether HAP affects MMP20 activity, we added different amount of HAP into the 96-well plate that contained MMP20 and its substrate, a quenched fluorescent peptide [Mca-Pro-Leu-Gly-Leu-Dap (Dnp)-Ala-Arg-NH_2_; Bachem, CA, USA] in 200 μL of MMP20 reaction buffer. The digestion was conducted at the enzyme/susbtrate ratio of 1:100. We measured hydrolysis of the substrate by monitoring fluorescence every 5 min for 1 h at 37°C with 320 nm excitation and 405 nm emission.

## Results

### MMP20 and KLK4 digestion progressively and extensively remove amelogenin from HAP

**Figure 1:**
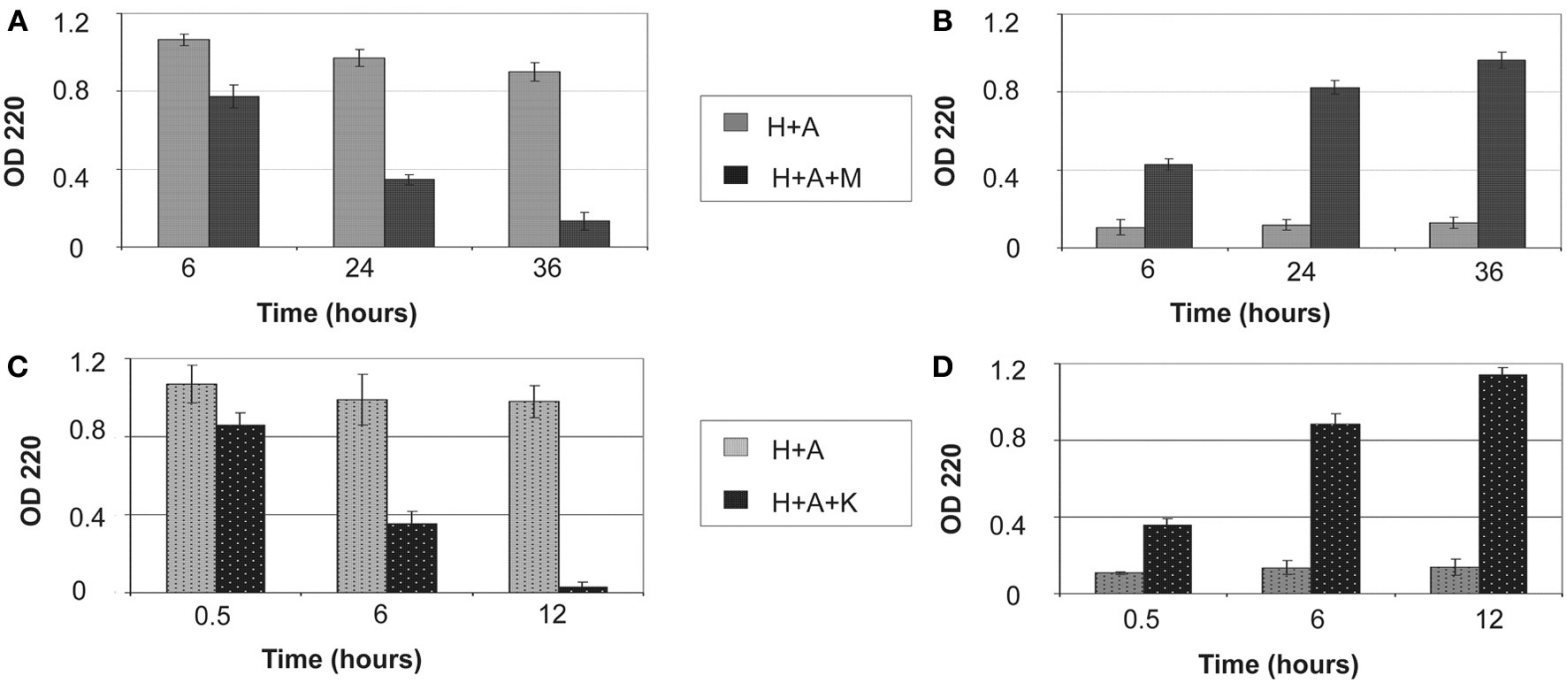
**Removal of bound amelogenin on HAP crystals by MMP-20 and KLK4 hydrolysis**. **(A)** Protein amounts remained on crystals and **(B)** released into surrounding supernatant at different time points during MMP20 digestion; **(C)** Protein amounts remained on crystals and **(D)** released into surrounding supernatant at different time points during KLK4 digestion. **H+A**, HAP+rh174; **H+A+M**, HAP+rh174+MMP-20; **H+A+K**, HAP+rh174+KLK4.

The amount of protein retained on HAP after digestion for various periods of time is illustrated in Figure 1. Both MMP20 and KLK4 hydrolysis could gradually remove the adsorbed amelogenin from HAP (Figures 1A,C) into the reaction buffer solution (Figures 1B,D). After 36 h of digestion by MMP20, only about 12% of amelogenin still remained on HAP. In comparison, the removal rate of adsorbed amelogenin was much faster by KLK4 digestion. Nearly 98% of amelogenin was released into the surrounding solution by merely 12-h processing. During the entire period of incubation with shaking, bound amelogenin could also be desorbed without digestion, but this loss appeared to be negligible compared to that observed in digested samples (Figures 1A,C). Because UV detection showed only a trace amount of MMP20 and KLK4 bound to HAP during digestion process, their effects on the measurement of amelogenin amount were not included from our analysis.

### Binding of amelogenin to HAP accelerated the rate of MMP20 or KLK4 hydrolysis

**Figure 2:**
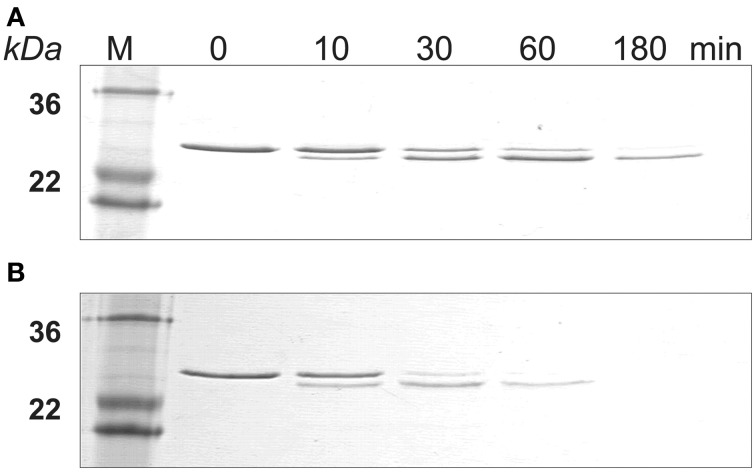
**MMP-20 digestion of amelogenin in solution and on HAP shown by SDS-PAGE. (A)** Amelogenin digestion in solution. **(B)** MMP-20 hydrolysis of amelogenin adsorbed on HAP. M, standard molecular weight.

The SDS-PAGE data showed that amelogenin bound to HAP was hydrolyzed by MMP20 faster than the proteins in solution (Figure 2). The amelogenin substrate on HAP progressively disappeared during the incubation. The intermediate digestion product at an apparent molecular weight of 23 kDa was further hydrolyzed by MMP20 into smaller fragments, which could not be detected by SDS-PAGE analysis. After 3 h of digestion, both the amelogenin at 25 kDa and derivative fragments at 23 kDa were almost completely degraded on HAP, but still partially remained in the samples without HAP.

**Figure 3:**
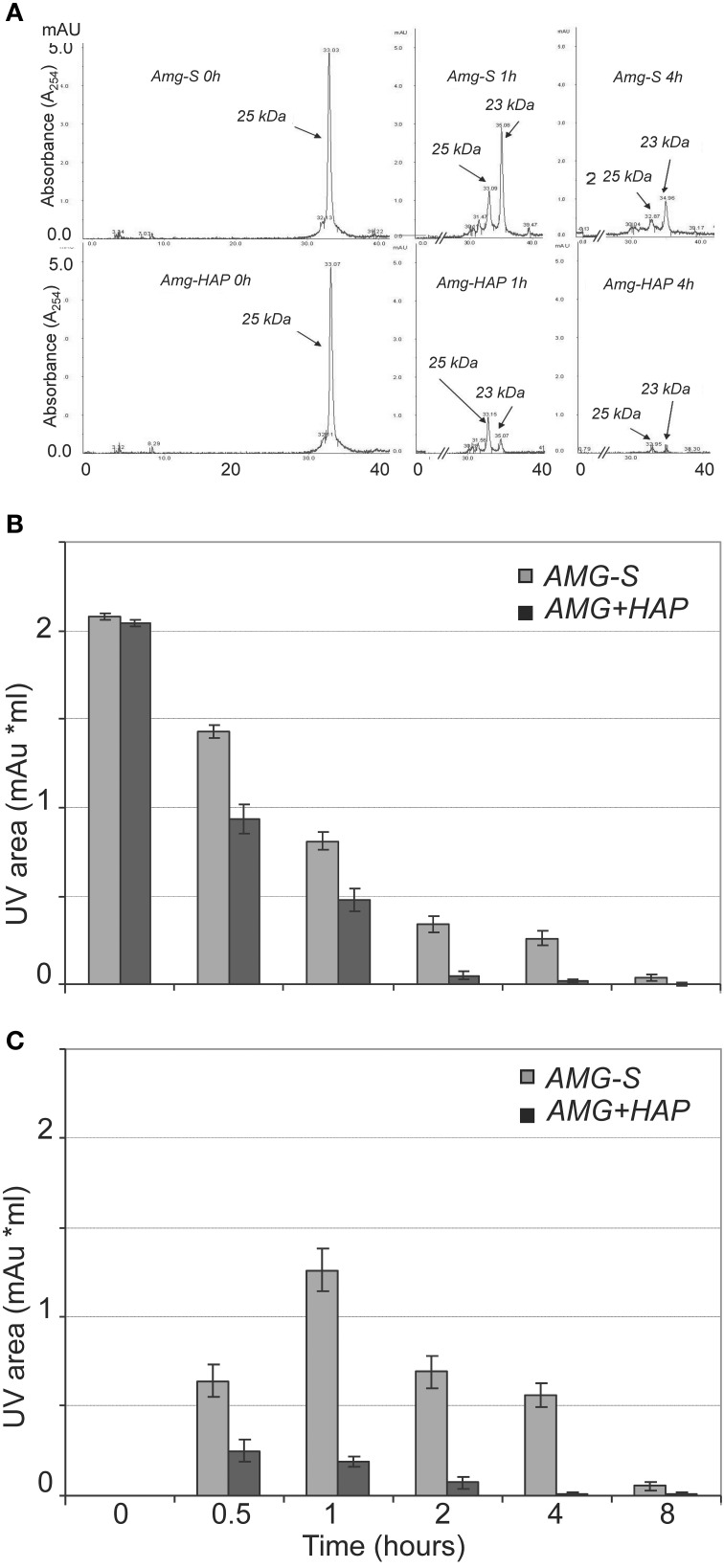
**MMP-20 hydrolysis of amelogenin in solution (AMG-S) and bound on HAP (AMG-HAP) were digested and quantified by HPLC. (A)** HPLC peaks of 25 and 23 kDa amelgenins after 1 and 4 h of digestion. **(B)** Quantified UV areas of 25 kDa amelogenin substrate after digestion. **(C)** Quantified UV areas of 23 kDa amelogenin derivative during digestion.

Further validation and comparison were achieved through HPLC analysis to observe the loss of the 25 kDa protein peak and concomitant generation of peptide peaks. The results of HPLC demonstrated that the digestion of amelogenin on HAP or in solution immediately produced one product peak (labeled “23 kDa” in Figure 3A). The corresponding HPLC fraction of this peak was collected and analyzed by MALDI TOF MS. The measured mass (m/z = 18,616) suggested that the 23 kDa band seen on SDS-PAGE corresponded to amelogenin truncated at P^164^/S (theoretical m/z = 18,615). The areas of individual peaks measured on HPLC traces before and after digestion were used to analyze the rate of hydrolysis. As shown in Figure 3B, the full-length amelogenin in solution was hydrolyzed significantly slower than the protein attached to the crystal surface. The same trend was observed for the 23 kDa proteolytic fragment, which was digested much slower in solution than in its HAP-bound form (Figure 3C). Interestingly, we note that the 23 kDa amelogenin fragment rapidly increases in the first hour of the digestion. This result indicates that MMP20 rapidly cleaves off amelogenin C-terminus, causing the accumulation of the truncated hydrophobic peptide in solution.

**Figure 4:**
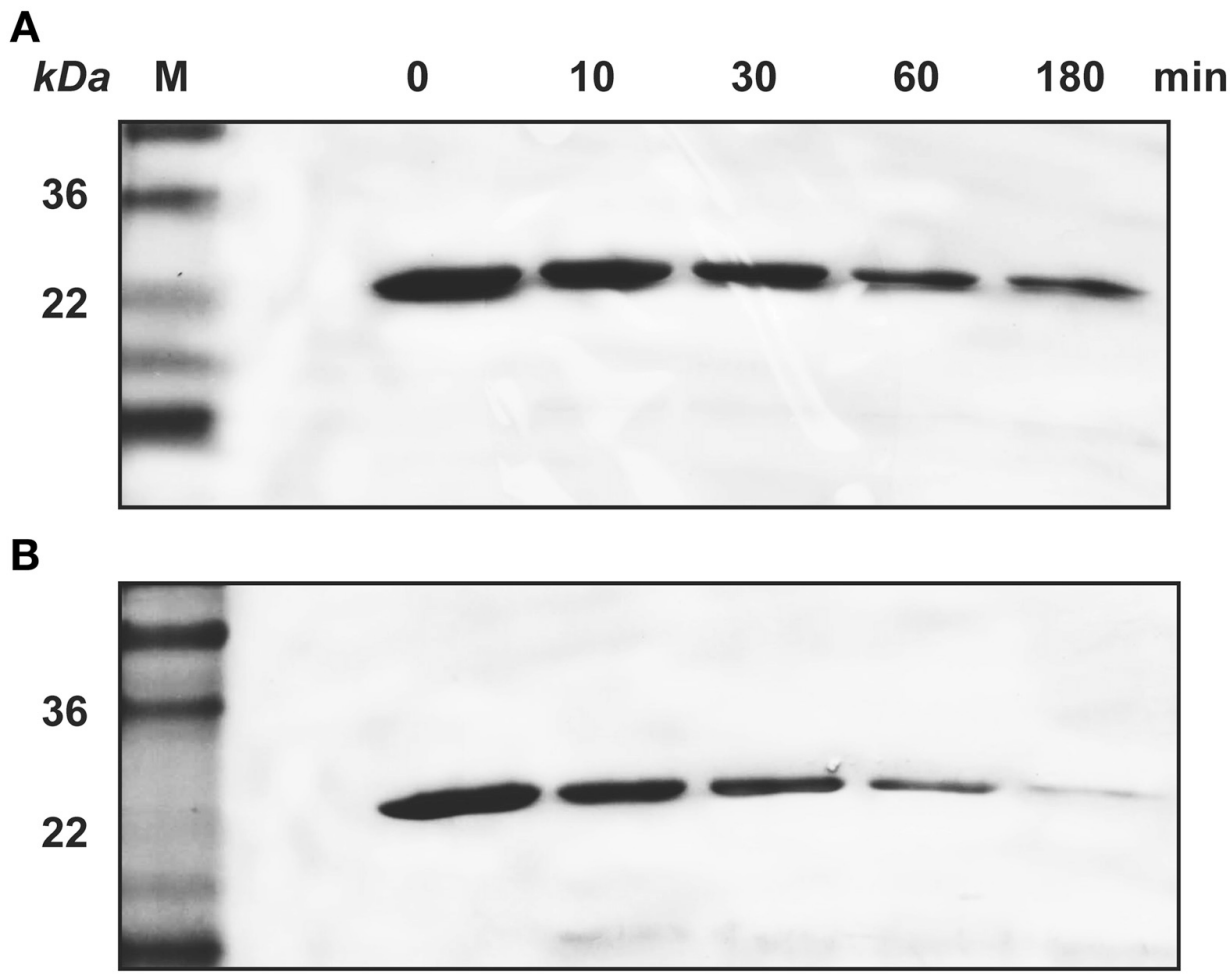
**KLK4 digestion of amelogenin in solution and on HAP shown by SDS-PAGE. (A)** Amelogenin digestion in solution. **(B)** KLK4 hydrolysis of amelogenin adsorbed on HAP. M, standard molecular weight.

Similarly, SDS-PAGE data showed that KLK4 hydrolyzed amelogenin much faster on HAP than that in solution (Figure 4). At the end of 180 min of KLK4 hydrolysis, there is much less HAP-bound 25 kDa amelogenin remained as compared with amelogenin digested in solution.

### Binding of amelogenin to HAP increased the accessible cleavage sites for MMP20/KLK4

Previous *in vitro* proteolysis experiments have shown that unbound amelogenin is cleaved by MMP20 primarily at its N- and C-termini (Fincham et al., 1981; Tanimoto et al., 2008a,b). To detect whether the adsorption to HAP affects amelogenin cleavage pattern by proteases, samples digested in the presence or absence of HAP were analyzed by LC-MALDI MS/MS. Table 1A lists the cleavage sites of MMP20 and KLK4 in amelogenins adsorbed on HAP and in solution, identified by mass spectrometric analyses. Amelogenin residing on HAP was cleaved by MMP20 at 54 sites, out of which 30 appeared to be unique for the HAP-bound substrate (Table 1B). Similarly, KLK4 produced more cleavages within amelogenin bound to HAP (78 sites), in comparison to samples without HAP (53 sites) (Table 1C). These results suggested that HAP indeed influences the cleavage pattern of adsorbed amelogenin and the larger number of utilized cleavage sites might thus explain an enhanced proteolysis after the proteins adsorb to HAP. In addition, interaction with HAP may also expose additional cleavage sites within the central region, the most hydrophobic part in amelogenin (Tables 1B,C).

**Table 1:** **The numbers of cleavage sites generated by MMP-20 and KLK4 in amelogenins digested on HAP and in solution**.

**Proteinases**	**Numbers of cleavage sites**	
	**Amelogenin on HAP**	**Amelogenin in solution**
**(A)**		
MMP20	55	25
KLK4	79	54
**(B) CLEAVAGE SITES IN HUMAN AMELOGENIN FOR MMP20**		
MPL^*^PPHPGHPGY/IN/F/S/YE/VLTP/LK/W/YQS^*^IRPPYP/S^*^Y^*^G^*^YEP^*^MGG^*^W/L^*^HH^*^Q^*^IIP^*^V^*^L^*^S^*^Q^*^QHPPTHT^*^LQPHHHIPVVP^*^A^*^Q^*^QP/VIPQQP^*^MMPVP^*^GQ/H/S^*^MTP^*^IQ/HHQPNLPPP^*^A/Q^*^QP/YQPQP/VQ^*^PQPHQP^*^MQPQPPVHP/MQ^*^P/L/PPQPPLPPMFP/MQ^*^PLPPMLP^*^DL/T/LEAWP/STDKTKREEVD		
**(C) CLEAVAGE SITES IN HUMAN AMELOGENIN FOR KLK4**		
MPLPPHPGHPG^*^Y/I^*^N/F/S^*^Y/E/V/LTP^*^L^*^K/W/Y/Q/S/IR/PPYPS/YGYEP/M/G/G/W/L/H/H/Q/IIPVLS/Q^*^QHPPT/H^*^T/L^*^Q^*^P^*^H/HHIP/VVP^*^A/QQP/VI/PQ/Q^*^PM^*^MPVPG^*^QH/S/MTP/IQ^*^H/H^*^QPN^*^LPPPA^*^QQPY^*^QPQP/VQPQPH^*^QP^*^M/QPQPP^*^VHP/M/Q^*^PL/PPQ^*^PPLPPM/FP/M/QP/L/PP/MLP/D/L/T^*^L/E/AW/PSTDKTK/REEVD		

The / in **(B)** and **(C)** indicates the MMP20 or KLK4 cleavage sites identical in both amelogenins adsorbed on HAP and in solution. The ^*^ indicates the cleavage sites of MMP20 or KLK4 unique for amelogenins adsorbed on HAP.

### Reduced MMP20 activity by bare HAP

**Figure 5:**
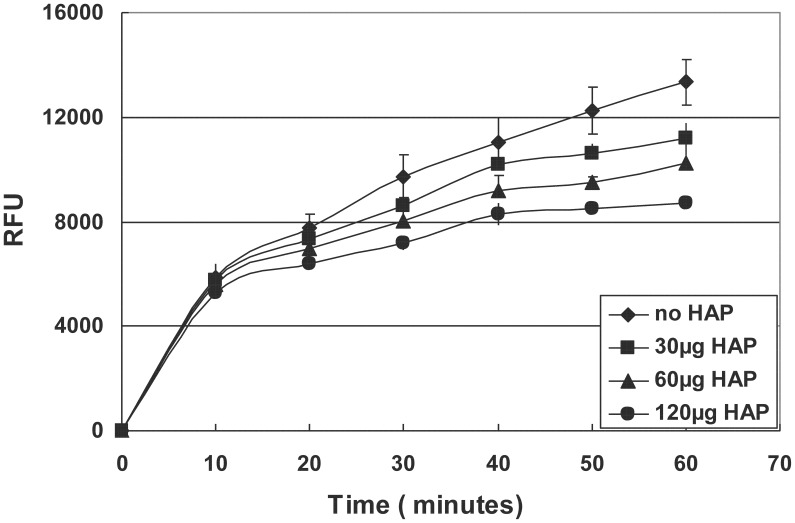
**Hydrolysis of quenched peptide by MMP20 in the presence of different amount of HAP**.

The quenched-peptide assay showed that the addition of bare HAP into the digestion reaction resulted in a significant decrease in the enzymatic activity of MMP20 in a dose-dependent manner (Figure 5).

## Discussion

The highly mineralized enamel crystals develop from a layer of enamel protein matrix that is predominated by amelogenins (>90%), which form special nanostructures to modulate crystal formation (Fincham and Moradian-Oldak, 1995; Fincham et al., 1999; Moradian-Oldak et al., 2001). The multilayer of amelogenin nanospheres in the matrix surrounding the thin and long crystals not only provide physical protection and mechanical support to these newly formed and extremely fragile crystals, but also modulate and inhibit their growth (Moradian-Oldak et al., 2001). During transition and maturation stages of enamel development, amelogenins are gradually processed by enamel proteinases, and mineralization simultaneously increases to form a fully mineralized enamel matrix (Fukae et al., 1998; Bartlett and Simmer, 1999; Li et al., 1999; Ryu et al., 1999, 2002; Simmer and Hu, 2002; Bartlett et al., 2003).

It has previously been evidenced that full-length amelogenin proteins strongly adsorb to apatite crystals and retard their further growth (Doi et al., 1984; Aoba and Moreno, 1987). Therefore, it is not difficult to understand that the removal of the proteins from matrix not only opens the space but also eliminates the possible inhibitory effects of amelogenin for enamel crystal growth. The question is which part of the matrix will be degraded and removed first, the proteins bound to crystals or unbound to appetite? If the amelogenins in the matrix (unbound to crystals) were digested faster or even at the same rates as those adhered to the crystal, the newly formed crystals with extreme thin and long structures would lose the protection and support. Consequently, they would be easy to collapse, break apart or fuse together. Therefore, the layers of amelogenin adsorbed on crystal surface need to be preferentially removed to open up the space for the growth of a new layer of crystals, while still having relatively intact surrounding protein matrix to support the thin and fragile nascent crystals.

The amelogenin-crystal interactions may contribute to this preferential degradation by proteinases. Proteins usually alter their conformation when bound to solid surfaces (Hlady and Buijs, 1996; Gray, 2004). In the recently published report by the Shaw group, they analyzed self-assembled amelogenin residing on single fluoroapatite (FAP) crystals, whose structures are the same as those of HAP crystals. They found that protein layer on the FAP surfaces were much thinner than the nanospheres originally present in solution. Their studies provide the strong evidence that amelogenin nanospheres undergo structural changes upon interacting with apatite surfaces (Tarasevich et al., 2009a,b). By using Fourier transform infrared (FTIR), the Beniash group also reported on the reduction in structural organization of amelogenin proteins upon their interactions with minerals at neutral pH (Beniash et al., 2012). In addition, it was shown that amelogenin nanospheres disassembled into oligomers and monomers when they adsorbed onto positively or negatively charged mica surfaces (Chen et al., 2012). The adsorption-induced conformational changes of the amelogenin and its assemblies can further alter their interactions with other enamel proteins, including proteinases (Smolarczyk et al., 2005; Sakiyama et al., 2006). The results of the present work clearly demonstrated that the binding of amelogenin to apatite crystals exerts significant effects on the proteolysis of the protein. Compared to the unbound proteins, the HAP-bound amelogenins were more rapidly hydrolyzed by both MMP20 and KLK4. Moreover, the additional proteinases-susceptible cleavage sites, primarily in the central region, were uncovered upon amelogenin binding to HAP. Previous studies suggested that the hydrophobic central region of amelogenins forms a dense core of nanosphere surrounded by the hydrophilic C-terminal tails in solution (Margolis et al., 2006). The buried central region is not readily accessible to the proteinases, and hence MMP20 hydrolysis of amelogenin occurs primarily at its termini. The interactions of HAP and amelogenins result in the disassembly of nanospheres into monomers (Chen et al., 2012), which may effectively exposes the central region of the protein. As a result, more cleavage sites become accessible and a higher rate of hydrolysis was observed.

The present study identified that MMP20 and KLK4 hydrolyzed amelogenin in solution at 25 and 54 cleavage sites respectively. These numbers are significantly higher than those previously reported (Nagano et al., 2008, 2009). In addition, we detected seven MMP20-susceptible cleavage sites within the sequence of tyrosine rich amelogenin peptide (TRAP). Previous studies indicated that TRAP was not susceptible to MMP20 hydrolysis (Nagano et al., 2008, 2009). The differences in experiment results among laboratories may result from several reasons. First, as we aimed at investigating the complete enzymatic degradation of amelogenin, beyond the initial generation of large proteolytic fragments, we used relatively higher enzyme-to-substrate ratios, longer incubation time and also replenished enzyme after 24 h of incubation. Second, in order to maintain similar conditions when comparing digestion of amelogenin in solution and bound to HAP, all digestion reactions utilized constant and vigorous shaking to keep HAP particles suspended in solution. The shaking procedure is very critical in these experiments, because HAP easily forms big clumps, especially after amelogenin adsorption. In addition, we used both amelogenin and proteinases from human species that is different from other reported ones, which may be another reason for these different cleavage activities.

Proteins and peptides usually inhibit the crystal growth by blocking the attaching sites and they should be completely or partially detached to facilitate the further crystal growth (De Yoreo and Vekilov, 2003; De Yoreo et al., 2007; Friddle et al., 2010, 2011). Our proteolytic experiments (Figure 1) showed that MMP20 or KLK4 hydrolysis of amelogenin resulted in gradual loss of its binding affinity to HAP and nearly complete release of the protein from HAP. Furthermore, we found that KLK4 digested amelogenin more efficiently and more completely than MMP20, demonstrating its major role in the clearance of amelogenin from enamel matrix at the maturation stage.

A previous publication reported that HAP reduced amelogenin hydrolysis by MMP20 and KLK4 (Sun et al., 2010), which seems to contradict our current finding at the first glance. However, after further comparisons, we found that the difference was due to the different experimental systems and techniques used in these two studies. In Sun's study, HAP was the last component to be added into the reaction mixture containing amelogenin and proteinases. In contrast, in our system, amelogenin was first adsorbed onto HAP in a pre-binding step. We used the pre-binding procedure to saturate the HAP surfaces with amelogenins to mimic the physiological and developmental conditions *in vivo*. As we have known, in the secretory stage, enamel matrix contains a large amount of amelogenins and the majority of these amelogenins remain intact or are only partially hydrolyzed by proteinases (Smith et al., 1989). The amelogenin protein matrix surrounding the enamel crystals will interact with the surface of HAP, which would alter the conformation of the bound protein and in turn affect protein hydrolysis and crystal growth. The result that emerged from the experiments that utilized a pre-binding step prior to proteolysis indicated that the adsorption-induced changes of amelogenin hydrolysis should mainly result from the interactions between amelogenin and HAP crystal surfaces, rather than from the proteinase-crystal interactions as shown in our quenched peptide experiment (Figure 5). In this experiment, different amounts of HAP were directly added into mixtures of MMP20 and its fluorescent substrate. The data showed dose-dependent inhibitory effects, indicating that the bare HAP can directly interact with MMP20 and inhibit its enzymatic activity.

**Figure 6:**
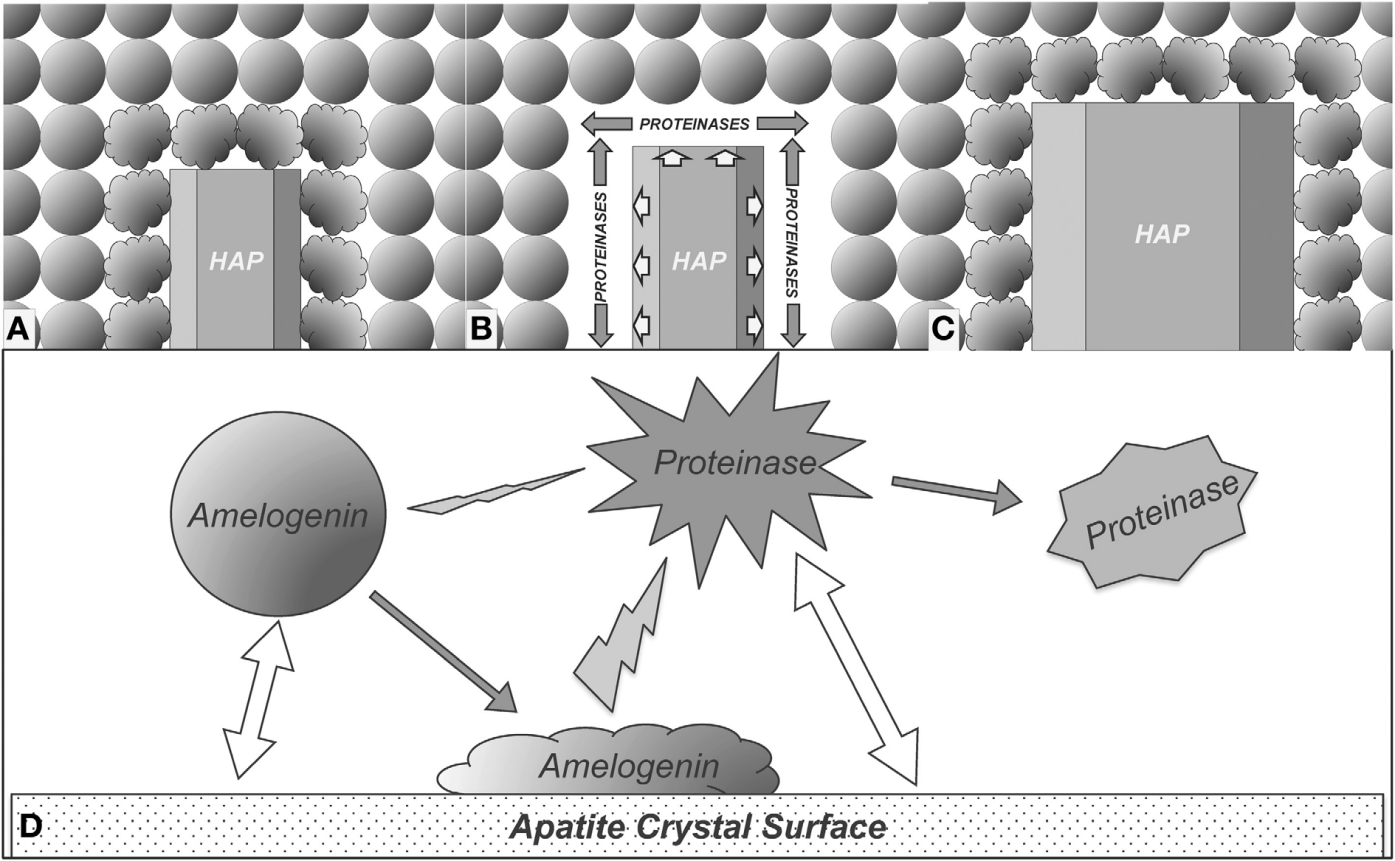
**Proposed model for enamel crystal growth guided by crystal-amelogenin-proteinase interactions. (A)** HAP crystal interacts with amelogenins and causes their structural changes; **(B)** The structural changes of adsorbed amelogenins result in preferential amelogenin degradation on crystal surface; **(C)** Removal of the amelogenin on the crystal surface releases the space for HAP crystal growth; **(D)** Summary of the proposed model of enamel crystal growth guided by crystal-amelogenin-proteinase interactions. Amelogenin interactions with crystal surface change its conformation, which increases its susceptibility to proteinase. The direct effect of proteinase-crystal interactions will change the structure of proteinase, showing inhibitory effects.

Based on the results obtained from this study, we propose a model to explain how the enamel crystal growth is mediated by interactions among crystal, amelogenin, and proteinases. As shown in Figure 6A, the amelogenins secreted into enamel matrix first bind to the nascent HAP crystals and the adsorption of amelogenins onto the crystal surface results in conformational changes of the bound proteins. The conformational changes may expose more cleavage sites to proteinases. As a result, cleavage of amelogenins by MMP20 or KLK4 is enhanced (Figure 6B). The preferential removal of amelogenins from the crystal surface opens up the space surrounding the crystals for their further growth (Figures 6C,D). When the nascent crystals growing in this new space come into contact with the next layer of amelogenin nanospheres, yet another cycle of the interaction-mediated preferential removal of bound amelogenin and crystal growth is initiated. The cycle of *binding-growth-digestion* repeats until the HAP crystals grow to fill up the entire enamel space during tooth enamel development.

### Conflict of interest statement

The authors declare that the research was conducted in the absence of any commercial or financial relationships that could be construed as a potential conflict of interest.
